# Siglec15 in blood system diseases: from bench to bedside

**DOI:** 10.3389/fimmu.2024.1490505

**Published:** 2024-12-04

**Authors:** Yujia Fan, Liangliang Sun, Juan He, Yuetong Chen, Hongli Ma, Haitao Ding

**Affiliations:** ^1^ Baotou Medical College of Inner Mongolia University of Science and Technology, Baotou, Inner Mongolia, China; ^2^ Clinical Laboratory Medicine Centre, Inner Mongolia Autonomous Region People’s Hospital, Hohhot, Inner Mongolia, China

**Keywords:** Siglec-15, immune checkpoint, osteoclasts, microbial infection, immunotherapy, hematological diseases

## Abstract

Inhibiting the PD-1/PD-L1 pathway using immunomodulators has demonstrated promising outcomes in clinics. Immunomodulators can effectively target immune checkpoints with a strong preference for the tumor microenvironment (TME). Besides, immunomodulators specifically target the recently discovered inhibitory immune checkpoint, sialic acid-binding immunoglobulin-like lectin (Siglec-15). Distinctive in its molecular composition, Siglec-15 has a unique molecular composition and been shown to be highly prevalent in numerous solid tumor tissues and tumor-associated macrophages (TAMs) in human subjects. Notably, Siglec-15 is up-regulated across various cancer types. As a result, Siglec-15 has attracted significant attention due to its exclusive nature concerning PD-L1 expression, suggesting its role in immune evasion in patients lacking PD-L1. Siglec-15 predominantly appears in certain populations and can promote tumor development by repressing T lymphocyte activation and proliferation, thereby facilitating tumor cell immune escape. Furthermore, Siglec-15 is implicated in osteoclast differentiation and bone remodeling, indicating that it is a promising target for next-generation cancer immunotherapies. Additionally, Siglec-15 can modulate immune responses to microbial infections. The current treatment strategies for hematological conditions predominantly include conventional intensive chemotherapy and transplantation methods. However, emerging immunotherapeutic approaches are increasingly recognized for their overall effectiveness, indicating that specific molecular targets should be identified. The expression of Siglec-15 within tumor cells may indicate a novel pathway for treating hematological malignancies. In this study, the biological attributes, expression patterns, and pathogenic mechanisms of Siglec-15 across various diseases were reviewed. The role of Siglec-15 in the pathogenesis and laboratory diagnosis of hematological disorders was also evaluated.

## 1 Introduction

Immune Checkpoint (IC) is a class of immunosuppressive molecules (1). Immunotherapy has garnered significant interest due to its minimal side effects, prolonged efficacy, and broad applicability across various cancer types (2). Immune checkpoint blockade (ICB) therapy interacts with ligands and receptors within the tumor microenvironment (TME), suppressing T cell activation, resulting in inappropriate activation of governing signaling paths in the immune response. This process hinders effective pro-inflammatory responses and diminishes anti-tumor immune effects (3). Notably, major advancements have been made in transitioning knowledge from biology to pharmaceuticals due to the discovery of CTLA-4 and the development of antibodies (4). Unlike previous immunotherapy approaches, anti-PD-1/PD-L1 treatments can regulate the immune feedback at the lump place, address immune deficiencies caused by tumor progression, and restore active tumor immunity (5). Therefore, studies should focus on mechanisms of immune evasion in tumors to understand effector T cell responses within tumor locations (6). PD-1/PD-L1 monoclonal antibodies have marked a significant milestone in cancer therapy, gaining approval for many tumors. This success is largely attributed to the selective expression of PD-L1, which is significantly suppressed within TME. Furthermore, PD-L1 improves patient response after tumor removal (7). Nevertheless, the total response rate is under optimal levels (less than 30%) because of the significant variability in tumors and the redundancy of mechanisms that allow immune evasion. Notably, most cancer patients demonstrate either main or gotten resistance (8). Wang et al. (9) identified a new immunomodulator, Siglec-15, using the Genome-Scale T-cell Activity Array (TCAA) TME. Siglec-15 can substantially suppress found antigen-specific T cell activity *in vitro* and *in vivo*. Siglec-15 can influence the activation of immune cells by interacting with sialylated pathogens (10). A recent study showed that immunotherapy can treat various solid tumors and hematologic cancers. Specifically, immune checkpoint inhibitors (ICIs) have been approved for treating Hodgkin lymphoma and primary mediastinal large B-cell lymphoma (11). The primary methods for treating hematological disorders include chemotherapy and transplantation. However, immunotherapy, recognized as a developing cancer treatment, has not yet assumed a significant position in this context. Siglec-15 functions as an immune checkpoint that effectively inhibits T cells, facilitating immune evasion by tumor cells. Additionally, Siglec-15 plays a distinct role in macrophage behavior and can reactivate inactive myeloid cells, indicating that Siglec-15 can treat hematological conditions.

**Figure 1:**
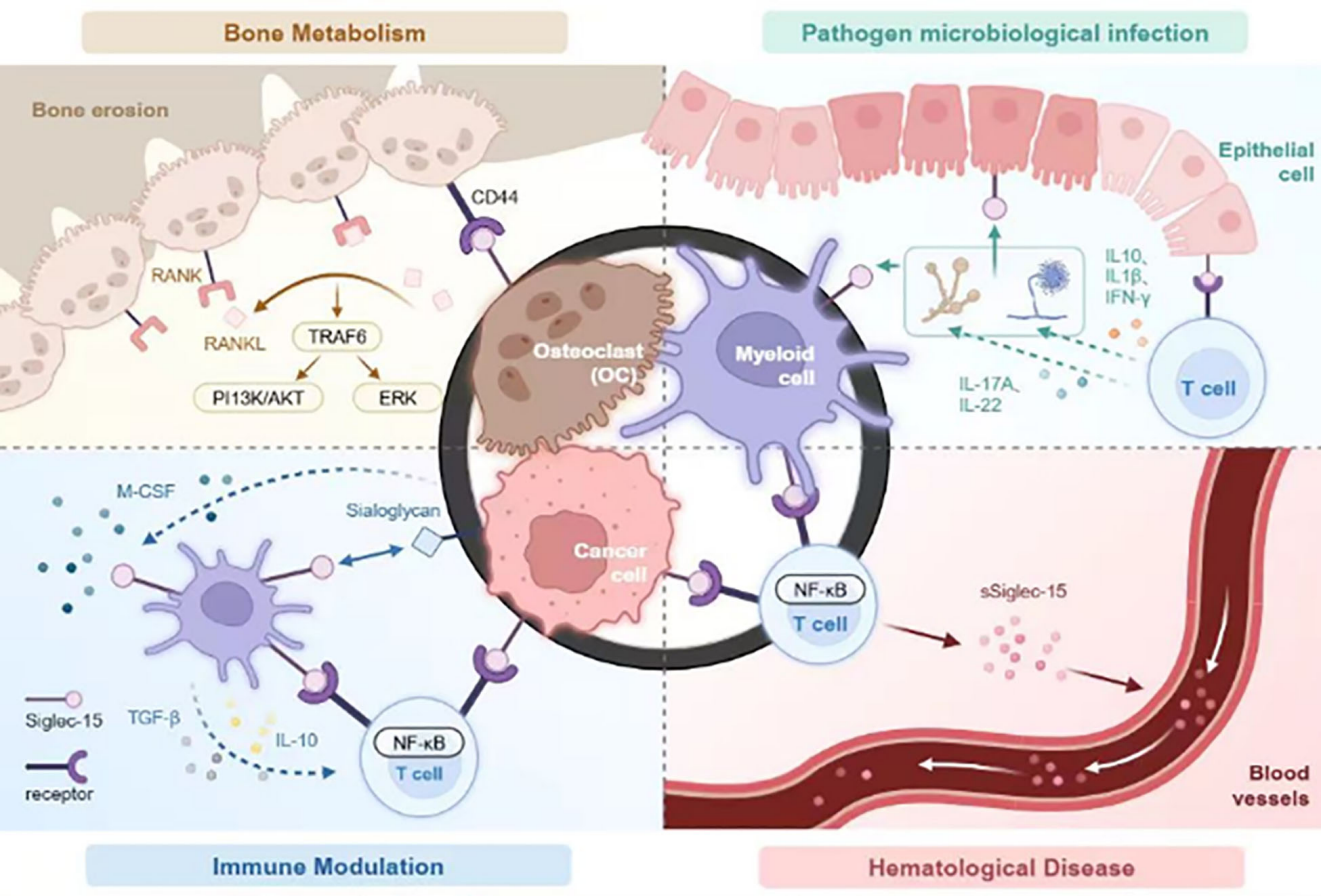
Siglec-15 affects four key areas: bone metabolism, modulates infections caused by pathogenic microorganisms, immune regulation, and the pathogenesis of hematologic diseases. In bone metabolism, Siglec-15 interacts with CD44 expressed on osteoclast precursors to stimulate the DAP12-SYK pathway and RANKL-RANK-TRAF6 signaling cascade, activating ERK and PI3K-AKT activation. It also been shown to be activated by microbial agents, thereby associating with unidentified receptors on T cells to regulate cytokine release and phagocytic activity to regulate immune responses. In immune functions, Siglec-15 expressed on tumor-associated macrophages and/or cancer cells interacts with unknown receptors on T cells, inhibiting T cell proliferation. Elevated levels of TGF-β and/or IL-10 further inhibits T cell activation. Studies have also demonstrated thatSiglec-15 is expressed on hematological tumor cells where is interacts with enigmatic receptors on T cells, modulating immune responses via the NF-kB signaling pathway. It exists as a soluble form in the bloodstream, contributing to diverse immune reactions in the body.

This paper provides a thorough analysis and summary of the biological features, expression patterns, and mechanisms of Siglec-15 across various diseases, including cancer, highlighting its potential for application in disease prevention, diagnosis, and treatment. Furthermore, the clinical use of Siglec-15 in managing patients with hematological diseases, including aspects of diagnosis, condition assessment, and treatment guidance, was evaluated (**Figure 1**).

## 2 Overview of Siglec-15

### 2.1 Molecular characterization of Siglec-15

The Sialic acid-binding immunoglobulin-like lectin (Siglec) family is the most extensive subgroup within the immunoglobulin superfamily (IGSF). Siglec family members play a critical role in the immune system by specifically recognizing sialic acid-containing glycans, which serve as their ligands. This recognition is essential for distinguishing between self and non-self entities. As a result, Siglecs significantly regulate and modulate immune responses (12). Increased tumor sialic acid content is related to various glycan species. Research has indicated that the proliferation rate of fibrosarcoma cells lacking sialic acid is slower than the fully sialylated cells of immunocompetent and radiation-naïve mice (13). This strongly suggests that the binding of Siglecs to elevated levels of sialic acid may facilitate tumor immune evasion (14). Besides, an increase in sialylation of tumor cells is correlated with metastasis and unfavorable outcomes in cancer patients (15). Siglecs are expressed on various leukocytes, primarily on hematopoietic cells (in the bone marrow and B cells) and non-hematopoietic cells (neurons) (16). Siglecs play a crucial role in numerous immune functions, including the modulation of inflammatory processes, leukocyte proliferation, interactions between hosts and microbes, and cancer-related immunity (17). Human Siglecs can be classified into two major groups based on sequence similarity. The first group consists of the relatively conserved CD22-related Siglecs, such as Siglec-15, and the second group includes CD33-related Siglecs, which exhibit various interspecific and intraspecific variations (17). Polymorphisms in the CD33 gene may lead to the development of human diseases. Furthermore, CD33-related Siglecs (CD33rSiglecs), which are present on leukocytes, can efficiently induce endocytosis and participate in innate immune responses, potentially indicating how pathogens may evade immune detection through their interactions with Siglecs (18).

Siglec-15 is a key member of the Siglec protein family, first identified in 2001 by Angata et al. The researchers discovered a specific DNA fragment within the genome that could code a protein resembling Siglecs, and provisionally named it as X (19). Subsequent research culminated in the formal designation of this protein as “Siglec-15” in 2007 (20). The gene encoding Siglec-15 is located on human chromosome 18q12.3, distinct from the gene cluster encoding other classical members of the CD33-related Siglec subfamily (found on chromosome 19q13.3-13.4). The protein has an approximate relative molecular mass of 37 kDa and is classified as a type I transmembrane protein. The protein structure comprises three key regions: an extracellular region that extends outside the cell, a cytoplasmic region that lies within the cell, and a transmembrane region that traverses the cell membrane. The extracellular binding domain contains an immunoglobulin variable region (IgV) and a type 2 constant region (IgC2). The IgV domain contains a conserved arginine (R143) motif, an important amino acid sequence essential for preferential adhesion to sialyl-Tn (Neu5Acα2-6GalNAc), which contains sialic acid residues (20). The cytoplasmic region includes a short cytoplasmic tail that lacks the typical ITIM-like motif, which is responsible for signal transduction inhibition. However, the transmembrane domain of Siglec-15 exhibits a conserved, positively charged lysine residue that interacts with the activating adaptor proteins DNAX activation protein of 12 kDa (DAP12) and DNAX activation protein of 10 kDa (DAP10) due to its specific configuration. DAP12 and DAP10 possess an immunoreceptor tyrosine activation motif (ITAM) (21). The proteins DAP12 and Spleen tyrosine kinase (Syk) within the DAP12-Syk signaling pathway play a crucial role in the transfer of intracellular signals through SHP-1/2, thereby initiating immunosuppressive responses (22).

The ionic interactions within the transmembrane domain mediate the interaction between Siglec-15 and DAP12, establishing a distinct signaling pathway that can modulate tumor-associated macrophage (TAM) polarization while minimizing excessive antagonism. Notably, most Siglec family members are characterized by ‘tandem repeats’ in their extracellular region, consisting of one IgV domain and several IgC2 domains. In contrast, Siglec-15 contains only a single IgV domain and a single IgC2 domain (12). This extracellular structural characteristic is common among B7 family members. Siglec-15 exhibits a protein sequence identity of 20%-30% with the B7 family. Research has shown that Siglec-15 and Programmed Cell Death 1 Ligand 1 (PD-L1), belonging to the B7 family, have about 30% amino acid sequence similarity based on sequence alignment (9).

The molecular characteristics have suggested that Siglec-15 may exert similar effects as immunomodulators from the B7 family. Additionally, PD-L1, also known as B7-H1, plays a role in the PD-1 immunosuppressive pathway. This finding indicates that Siglec-15 possesses unique attributes that can inhibit immune escape. Notably, Siglec-15 is the only conserved Siglec throughout vertebrate evolution, with similar functions as B7 family members in regulating the immune response (20). The architecture of Siglec-15 is both distinctive and intricate, which sets it apart within the Siglecs family. However, the biological properties of Siglec-15 warrant further exploration.

### 2.2 Distribution and localization of Siglec-15

Angata et al. (20) discovered that homologues of Siglec-15 are present in fish, indicating that the homologous sequences of zebrafish and humans remain largely intact. This finding suggests that the paralogues of Siglec-15 exist across a diverse range of vertebrate lineages. Furthermore, the gene sequence for Siglec-15 has been identified in nearly all vertebrates throughout vertebrate evolution (17). Notably, no other known Siglec gene associated with the immune system has demonstrated such a conserved expression over the same evolutionary timeframe. Therefore, Siglec-15 can serve an important and conserved regulatory function within the immune systems of vertebrates.

Siglec-15 is primarily found on the plasma membrane layer of multinucleated osteoclasts and cytoplasmic regions within multinucleated cells, forming actin rings. Although Siglec-15 is a kind I transmembrane healthy protein, it is situated in the cytoplasm instead of on the membranes of macrophages or dendritic cells. Angata et al. (20) proposed that the visibility of a series concept (YENL) in Siglec-15 facilitates its movement into the cytoplasm, adhering to the recognized endocytic motif. Furthermore, they showed that endocytosis and the processes of vesicle trafficking play an essential role in bone resorption, osteoclast development, and the preservation of cell polarity. Similar to other transmembrane proteins that can be directed to the membrane, this motif may support the transport of proteins through the endosomal system, leading to their localization in the Golgi apparatus unless they are inherently stable on the membrane. Interestingly, Pillsbury et al. (23) showed that NFκB influences the localization and expression of Siglec-15 within cells. Specifically, NFκB activation by PMA reduces Siglec-15 localization within the Golgi apparatus after 24 hours while also enhancing its presence on the surface of B-ALL cells. This observation suggests that the activation of nuclear factor κB facilitates the increase in surface localization of Siglec-15. These findings imply that Siglec-15 can mediate the mechanisms related to endocytosis. Besides, Siglec-15 exerts an immunosuppressive effect via specific motifs by interacting with its ligands on immune cell surfaces, thus mediating inhibitory signals after transmembrane transport to the cell membrane.

### 2.3 Progress in the study of Siglec-15 ligands

Angata et al. (20) previously demonstrated that Siglec-15 is expressed in macrophages located in the paracortical and medullary sinus regions of the spleen’s marginal zone and lymphoid ganglia, where it binds to its specific ligand, Salyl-Tn. This ligand is widely expressed across numerous tumor cell types. Takamiya et al. (24) discovered that Siglec-15 enhances the secretion of TGF-β in tumor-associated macrophages (TAMs) by identifying sTn antigens associated with tumors. This suggests that Siglec-15 may influence the TME in macrophages through a TGF-β-mediated signaling pathway. Salyl-Tn is recognized as a potential high-affinity ligand for Siglec-15. Gavuthamid et al. (25) recently identified multiple sialylated carbohydrate structures that are well characterized and bound by Siglec-15, including DSMFLNH (disialylactosyl-N-hexasaccharide), LSTb (sialylactose-N-tetrasaccharide b), and DSLNT (disialylactose-N-tetrasaccharide). Some of these structures may function as natural ligands for Siglec-15. Chang et al. (26) achieved shRNA-mediated knockdown of CD44 in an osteoclast precursor model using the RAW264.7 cell line through the proximity labeling technique and indicated a reduction in RANKL-induced osteoclast activity and Siglec-15 binding. Furthermore, this research demonstrated that CD44 has high expression levels in various cancer cells and tumor-associated macrophages, suggesting that CD44 can act as a key ligand for Siglec-15 within TME. Lenza et al. (27) elucidated the crystal framework of Siglec-15 and identified its binding epitope through co-crystallization experiments involving an anti-Siglec-15 blocking antibody. They employed saturation transfer differential nuclear magnetic resonance (STD-NMR) spectroscopy and molecular dynamics simulations to investigate the binding interactions of Siglec-15 with α(2,3)- and α(2,6)-linked sialic acids, as well as the sialic acid Tn (STn) glycoform associated with cancer. Their findings demonstrated that the binding of Siglec-15 to T cells lacking sTn expression depends on the presence of α(2,3)- and α(2,6)-linked sialoglycoproteins. Additionally, they discovered that leukocyte integrin CD11b, belonging to the CD11 antigen-like family, acts as a binding partner for Siglec-15 on human T cells. Antibodies targeting Siglec-15 can bind to CD11b on the surface of T cells, thus significantly reducing Siglec-15 binding when CD11b is blocked. Conversely, CD11b/CD18 overexpression enhances the binding of Siglec-15 to T cells, a process dependent on the sialic acid binding domain of Siglec-15 (27).

However, there is no appropriate ligand for Siglec-15. Therefore, future research should uncover sugar chains that bind to Siglec-15 with high specificity and robust affinity through chemobiological techniques for structural modification and evaluating the structure-activity relationship. This approach may lead to the discovery of Siglec-15 ligands and inhibitors with strong specificity and affinity.

## 3 The role of Siglec-15 in bone metabolism

**Figure 2:**
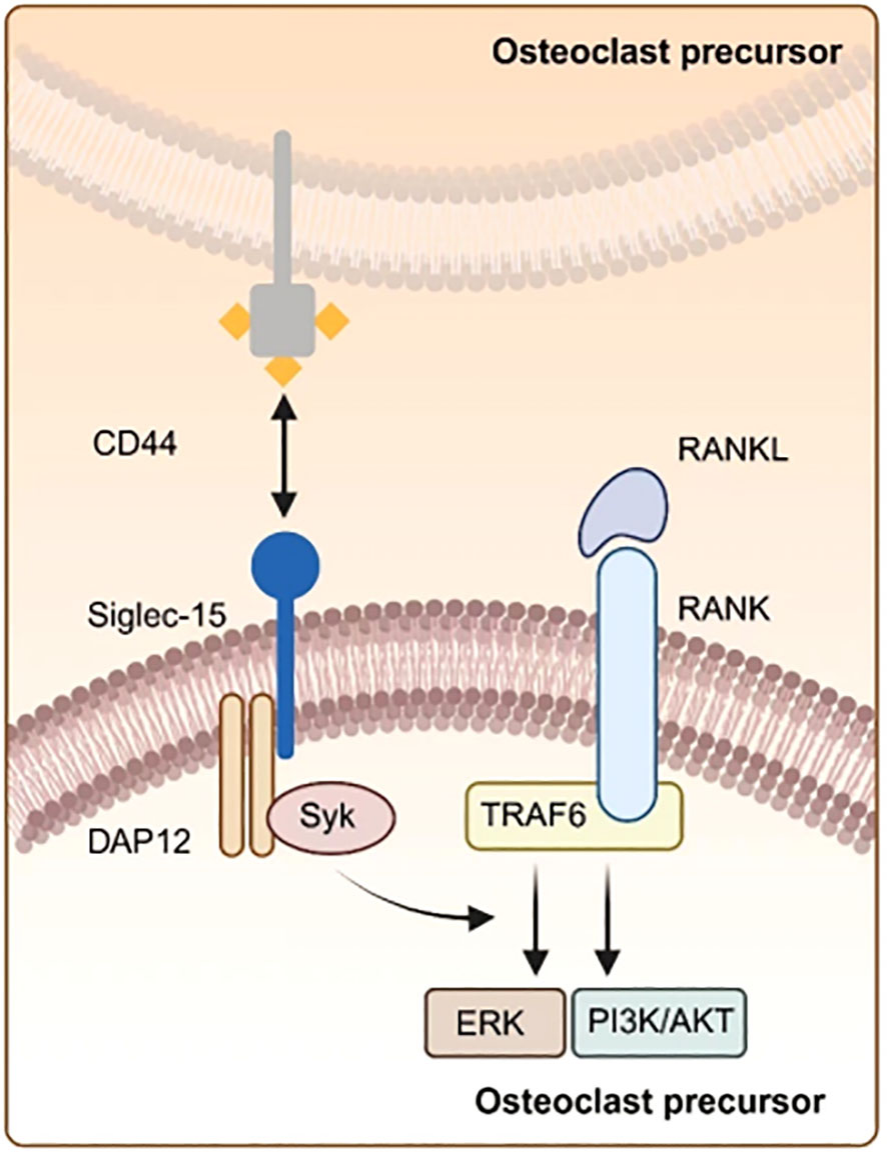
Illustration of osteoclasts differentiation. Siglec-15 can bind CD44 on osteoclast precursors via the DAP 12-SYK signaling pathway, which interacts with the RANKL-RANK-TRAF 6 pathway, thereby amplifying the expression of downstream signaling molecules like ERK and PI3K-AKT.

Myeloid multinucleated cells, also known as osteoclasts(OCs), are crucial for bone resorption and remodeling and play an important function in the upkeep of bone mass and calcium balance. Hiruma et al. discovered that Siglec-15 is significantly associated with OCs differentiation. Besides, they showed that the expression of Siglec-15 is significantly increased in both gigantic cell lumps of bone (GCT) and typical osteoclasts. The differentiation function of OCs is effectively suppressed in both mice and humans after the application of polyclonal antibodies targeting Siglec-15 (28). OCs generation is reliant on the signaling pathway involving RANKL, RANK, and TRAF6. Moreover, OCS differentiation is primarily initiated through the interaction between RANK ligand (RANKL) and the NF-κB receptor activator present on OCs precursors (RANK) within the NF-κB signaling cascade (29). RANKL is produced by OCs. The Ishida-Kitagawa team previously demonstrated (30) that the expression of activated T cell nuclear factor 2/c1 (NFAT2 or NFATc1), induced by RANKL, is crucial for the development of functional and multinucleated OCs. Furthermore, Immunoreceptor tyrosine activating motif (ITAM) is crucial for NFATc1 phosphorylation. Besides, RANKL/RANK system plays a role in physiological OCs formation via ITAM signaling. Additionally, OCS maturation facilitates accessory signaling and activation of the phospholipase Cγ (PLCγ) calcium pathway through DAP12 and FcRγ, which promotes NFATc1 expression. Finally, the DAP12-related immunoreceptor (DAR) Siglec-15, influenced by NFATc1, modulates OCs differentiation through the signaling pathway of RANKL-RANK-NFAT2 (31). The two signaling pathways operate concurrently. Yoshiharu et al. discovered that Siglec-15 is up-regulated in OCs following stimulation with receptor activator of nuclear factor-KB (RANKL) ligands (28). Additionally, Siglec-15 activates Syk via Lys-272 within the Asp-52-linked Siglec-15/DAP12 complex, which facilitates the phosphorylation of essential signaling molecules, including extracellular signal-regulated kinase (ERK), protein kinase B (AKT), and phosphatidylinositol 3-kinase (PI3K). These molecules function downstream of the RANKL-RANK-TRAF6 cascade (32, 33). The study indicated that inhibition of the PI3K or Akt pathways may affect the development of actin rings in OCS. Notably, OCS from mice lacking Siglec-15 cannot form actin rings (34). These findings imply that Siglec-15 can positively regulate OCs activity in bone resorption by modulating cytoskeletal organization and influencing OCs differentiation through the PI3K/Akt and ERK pathways (**Figure 2**).

While Siglec-15 is crucial for physiological bone remodeling through the regulation of RANKL signaling, its role in pathological bone loss associated with various osteoclastogenic factors that contribute to excessive osteoclastogenesis is unclear (35). Furthermore, studies involving transgenic mice have confirmed that Siglec-15 influences OCs. Hiruma et al. investigated the function of Siglec-15 during OCs formation and bone development using Siglec-15 ^−/−^ mice and discovered that the mice exhibited mild osteopetrosis and increased bone mass (36). The Kameda researchers also showed that bone mass increases in mice not expressing Siglec-15. Earlier studies also indicated that the disruption of OCs development increases bone mass (32). Stuible et al. revealed that inhibiting OCs activity using a monoclonal antibody against Siglec-15 can decrease bone resorption while simultaneously increasing bone mass *in vivo*. This finding indicates that targeting Siglec-15 with antibodies is an alternative treatment option for osteolytic diseases (33). Ishida-Kitagawa et al. discovered that the inhibition of OCs differentiation and bone resorption can be achieved through either the knockdown of Siglec-15 using shRNA or the application of a polyclonal antibody targeting Siglec-15 (33). Nonetheless, Siglec-15 knockdown does not cause significant physical anomalies in mice, except for minor osteopetrosis (36). Moreover, monoclonal antibodies against Siglec-15 can enhance bone mass while not adversely affecting bone growth (37). Siglec-15 in OCs mediates various conditions, including boosted bone traction by OCs and the metastasis of bone cancer cells. Additionally, disorders related to bone metabolism are related to various diseases, such as rheumatoid arthritis, which causes bone erosion and postmenopausal osteoporosis (38). The inhibition of Siglec 15 can lessen periarticular bone loss associated with murine rheumatoid arthritis in a computer mouse version of antigen-induced arthritis (AIA) (39). Similarly, research demonstrated that the blockade of Siglec-15 significantly decreases bone loss within a K/BxN serum transfer model (40).

Siglec-15 promotes OCs differentiation associated with estrogen deficiency in menopause-related osteoporosis. Furthermore, Siglec-15 inhibition can decrease bone loss *in vivo*, suggesting its potential as a therapeutic target for osteoporosis in postmenopausal individuals (35). Glucocorticoid-induced osteoporosis (GIO) significantly promotes secondary osteoporosis. A research with juvenile rat models of GIO revealed that the number of mature OCs reduces after Siglec-15 treatment while leaving osteoclast precursors unaffected. Importantly, this antiresorptive intervention does not negatively impact the development of long bones or the presence of normal OCs within the primary cavernous body. Kobayashi team also indicated that double knockout mice lacking both Siglec-15 and FcRγ exhibit dwarfism. The underlying pathological mechanism disrupts the differentiation and maturation of OCs in both the growth plate and primary spongiosa, thereby hindering bone growth and adversely affecting normal bone development. This suggests that Siglec-15 may facilitate bone resorption primarily through the regulation of the cytoskeletal structure in OCs (34).

In conclusion, the role of Siglec-15 in the field of bone biology requires further exploration. Nonetheless, efforts to target Siglec-15 for osteoporosis treatment have also progressed to clinical development (33). Therefore, further research is needed to provide new insights for designing clinical therapies for bone diseases.

## 4 Role of Siglec-15 in pathogenic microbial infection

### 4.1 Important immune response role of Siglec-15 in RVVC

The Siglec family members have distinct roles in the recognition of microbial pathogens and interacting with host immune defenses. This variation is due to the communication occurring between Siglecs on neutrophils or monocytes and sialylated pathogens (41). *Candida vaginitis* is commonly diagnosed using Siglec family members, with about 8% of females worldwide experiencing recurrent vulvovaginal candidiasis (RVVC) (42, 43). Jaeger et al. (10) indicated that Siglec-15 promotes the development of RVVC through a combination of genomic analyses and immunological investigations. A specific single nucleotide polymorphism (rs2919643 C, Phe273Leu) in the Siglec-15 gene represents a risk allele associated with RVVC susceptibility in individuals with RVVC. Furthermore, Candida enhances the expression of Siglec-15 in human peripheral blood mononuclear cells (PBMCs) and human genital epithelial cell lines after incubation with *C. albicans*. Studies have shown that Siglec-15 expression increases in human myeloid cells, human genital epithelial cell lines, and mouse vaginal epithelial cells in response to *C. albicans*, especially in myeloid cells (44). Earlier research indicated that sialic acid is present on the surface of C. *albicans* (44). This finding suggests that C. *albicans* may promote the expression of Siglec-15 in myeloid (or epithelial) cells, thus influencing T-cell function. Patients with this variant exhibit elevated levels of Th17-related cytokines, such as IL-17A and IL-22, in PBMCs after Candida stimulation. This polymorphic variant increases the expression of IL1B and NLRP3 in HeLa cells Candida stimulation *in vitro*. Furthermore, Siglec-15 expression is up-regulated on the vaginal surface of mice during infections. The inhibition of Siglec-15 through small interfering RNA (siRNA) increases Candida burden, alterations in inflammatory cytokine levels, and a rise in neutrophil count in an *in vivo* mouse infection model. Phe273 is located near Lys274, which interacts with DAP12 in the transmembrane segment of Siglec-15, indicating that this polymorphism may influence the signaling pathway associated with Siglec-15. These results enhance the understanding of the underlying mechanisms involved in vulvovaginal candidiasis, suggesting novel potential treatment strategies. Siglec-15 plays a crucial role in the host’s immune response to Candida infections since it affects cytokine production and the clearance of Candida, thereby modulating the immune response against these infections. This investigation provides new insights into the pathophysiology of RVVC and identifies potential therapeutic targets for managing the condition. Additionally, influenza-associated pulmonary aspergillosis (IAPA) is primarily triggered by the fungus Aspergillus fumigatus (44).

### 4.2 Significance of Siglec-15 in IAPA

**Figure 3:**
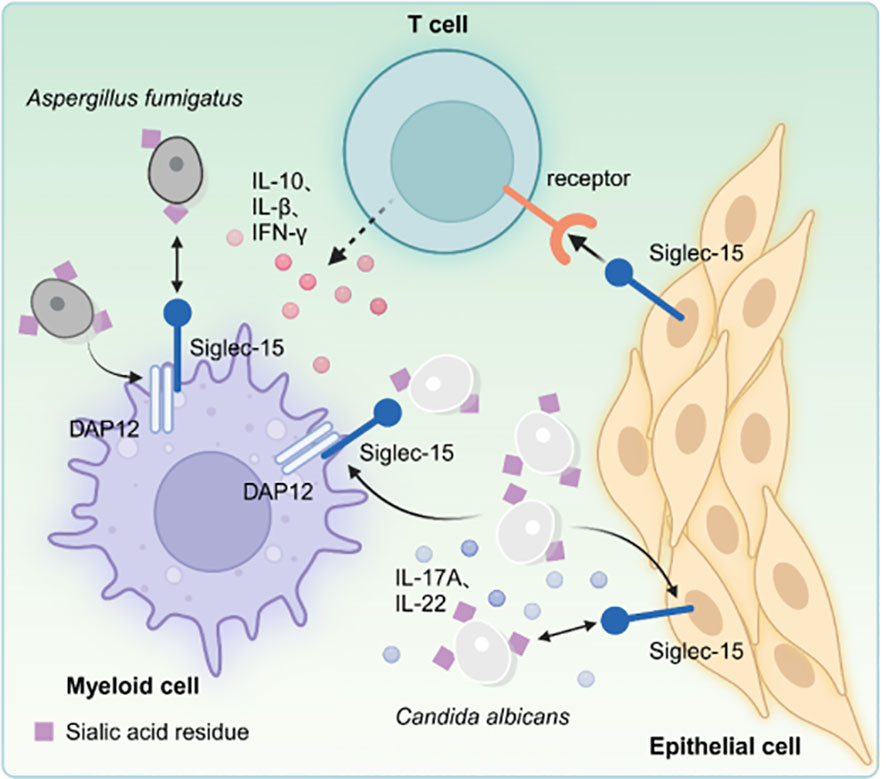
Microbial pathogens can activate the Siglec-15 expressed on bone marrow and/or epithelial cells, which in turn binds to unidentified receptors on T cells, inhibiting T cell activation. Moreover, the interaction between Candida and Siglec-15 increase the production of Th17-associated cytokines, including IL-17A and IL-22, in peripheral blood PBMCs. Genetic silencing of Siglec-15 results in suppression of IL-10 and TGF-β expression, while individuals with genetic polymorphisms exhibit decreased production of cytokines IL-1β and IFN-γ following exposure to *A. fumigatus* infection.

The occurrence rate of IAPA is about 20%, with a mortality rate exceeding 50% in cases of severe influenza 5% in intensive care unit (ICU) patients, and about 20% during influenza outbreaks (45). Dewi et al. (46) observed that PBMCs stimulation with A. *fumigatus*, significantly up-regulates in Siglec-15 after 24 hours. PBMCs protein can bind to sialic acid deposits found in the cell wall surface of A. *fumigatus*, suggesting that Siglec-15 may play a role in the host’s protective mechanisms against Aspergillus. Additionally, individuals with a specific polymorphism in the Siglec-15 gene (rs2919643) demonstrate a reduced cytokine response, particularly IL-1β and IFN-γ, following Aspergillus infection. Siglec-15 silencing and treatment with neuraminidase inhibitors, such as oseltamivir, can diminish the capacity of PBMCs cells to kill Aspergillus. Although Siglec-15 silencing and treatment with neuraminidase inhibitors do not significantly affect the production of TNF-α, the treatments markedly reduce TGF-β expression. This was the first to highlight the significance of neuraminidase and Siglec-15 in pulmonary aspergillosis, providing new insights into the disease’s pathogenesis. Furthermore, another study indicated that Siglec-15 polymorphism (rs61104666 A, a synonymous substitution at Glu292) is strongly correlated with pulmonary tuberculosis (47). However, further studies should assess the impact of Siglec-15 polymorphisms on the progression of the disease (**Figure 3**).

## 5 Immunomodulation of Siglec-15 in cancer

### 5.1 Tumor immunity research of Siglec-15

**Figure 4:**
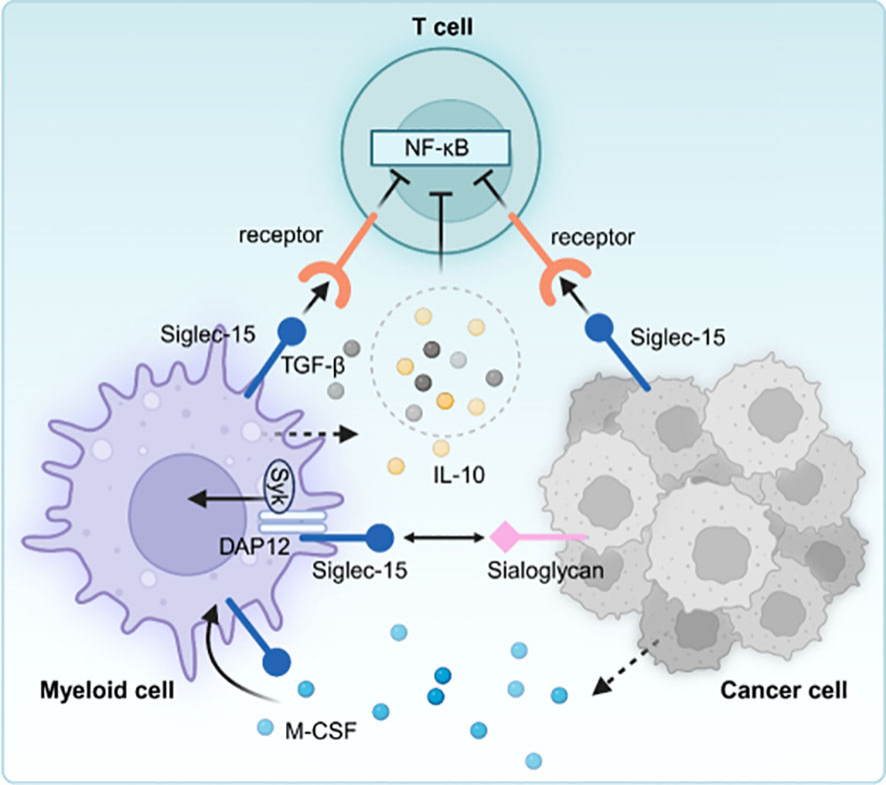
Siglec-15 is expressed on tumor-associated macrophages and/or cancer cells, interacts with unidentified receptors on T cells, decreasing the activity of T cells. Moreover, increased TGF-β and/or IL-10 further inhibits T cell activation in the tumor microenvironment.

The overexpression of the B7-H1 (PD-L1) molecule is a key immune evasion strategy within the TME of specific cancer patients. Notably, blocking the interaction between B7-H1 and PD-1 using antibodies can restore typical immune function with minimal adverse effects. Although Siglec-15 is exclusively expressed in certain myeloid cells, it is significantly up-regulated in human tumor cells and myeloid cells that infiltrate tumors. The mutual exclusion of Siglec-15 and B7-H1 is partly caused by M-CSF stimulation and the downregulation of IFN-γ (9). Research has demonstrated that Siglec-15 suppresses antigen-specific responses of T lymphocytes *in vitro* and *in vivo*. Siglec-15, as a suppressor molecule for T lymphocytes, is induced during tumor progression and immune evasion, exhibiting high expression levels within TME. Siglec-15 regulates T lymphocyte activity. The deletion of the Siglec-15 gene or its inhibition through antibodies in certain cancer models can transform the immunosuppressive TME into one that favors an inflammatory response while maintaining overall body homeostasis (**Figure 4**).

Wang et al. (9) introduced a high-throughput functional screening technology [genome-scale T lymphocyte activity array (TCCA)], which can effectively identify T lymphocyte activity *in vitro* through a cell surface regulator. The research confirmed that Siglec-15 is an immunosuppressive molecule for T lymphocytes in relation to macrophages. Initially, the team discovered that HEK-293T cells expressing Siglec-15 induce a suppressive effect on Jurkat T cells via the NF-κB pathway. Furthermore, they found that the soluble form of Siglec-15 can significantly reduce the expansion of human T cells induced by anti-CD3 (OKT3). Additionally, Siglec-15 hinders computer mouse T-cell expansion, cytokine release, and cytotoxic functions in artificial antigen-presenting cells (APCs). These findings demonstrate that Siglec-15 exhibits ligand-like properties by utilizing unidentified receptors to convey inhibitory signals that suppress T cell function and activity. The influence of Siglec-15 on T cells was later verified *in vivo* through a computer mouse design of autoimmune encephalomyelitis (EAE). Results showed that both Siglec-15-deficient mice and those given a fusion protein containing the extracellular domain of Siglec-15 experienced significantly worsened EAE, accompanied by markedly heightened T cell actions. Wang et al. examined the influence of Siglec-15 on tumor immunity employing B16-GMCSF (PD-1-insensitive) and GL261 (PD-1-sensitive) mouse models and indicated that: First, S15KO mice exhibited slower lump development and a considerable increase in the population of CD8+ T lymphocytes and NK cells in the B16-GMCSF model. Second, S15KO mice also demonstrated reduced tumor growth, a significantly enhanced response of tumor-specific CD8+ T lymphocytes, and an extension of survival in the mice in the GL261 model. The researchers showed that Siglec-15 inhibits immune actions against lumps, primarily by modulating tumor-associated macrophages. Furthermore, a monoclonal antibody targeting Siglec-15 was developed utilizing mixed tumor technology, preventing the interaction between Siglec-15 and T lymphocytes, thereby enhancing anti-tumor immunity. The research also indicated that the host’s macrophages and cancer cells can subdue T-cell anti-tumor resistance via Siglec-15. The Siglec-15 monoclonal antibody enhanced the activity of PD-1 knockout T cells and displayed synergistic immunostimulatory effects and anti-PD-1 antibodies on T cells in both artificial insemination and *in vivo* settings. Furthermore, the researchers noted that Siglec-15 can influence antigen-specific T-cell responses. Besides, the mice lacking Siglec-15 exhibited greater expansion of OT-I T cells in the blood and spleen following OVA peptide stimulation, similar to the phenotype observed in PD-L1 knockout mice. IL-10 may play an essential role in this mechanism, as evidenced by the reduced serum levels of IL-10 in Siglec-15-deficient mice compared with wild-type mice. Additionally, anti-IL-10 monoclonal antibodies eliminated the variation in OT-I T cell development in wild-type and Siglec-15-deficient mice. These findings indicate that specific monoclonal antibodies can treat tumors by inhibiting the effects of Siglec-15, particularly when combined with anti-PD-1 therapies for enhanced effectiveness. However, the immunosuppressive mechanisms mediated by Siglec-15 act independently of the PD-L1 pathway, suggesting that Siglec-15 and PD-L1 engage different signaling paths and maintain separate action mechanisms in immune regulation. Therefore, targeting Siglec-15 may offer immunotherapeutic approach for individuals with resistance to anti-PD-1 therapies.

### 5.2 Pan-cancer analysis of Siglec-15

The mRNA of Siglec-15 can be enhanced in various human cancer cells, including colon, endometrioid, and thyroid cancer cells (**Table 1**). Moreover, Siglec-15 mRNA is significantly up-regulated in bladder, renal, lung, and liver cancers compared with the corresponding normal tissues (9). Siglec-15 mRNA levels are significantly low in most normal tissues and immune cell subsets compared with tumor-associated tumor cells and macrophages (61). A seminal study of Siglec-15 in non-small cell lung cancer (NSCLC) highlighted that this molecule hinders antigen-specific T-cell activation (9). Liang et al. (62) demonstrated that Siglec-15 promotes the progression of NSCLC, development of spinal metastasis, and suppression of T-cells. CD8 + T cell-mediated cytotoxicity plays a crucial role in the immune response against breast cancer (63). Notably, the overall response rate in patients with advanced breast cancer using PD-1 blockers is only 18.5% (64, 65). A study showed that Siglec-15 protein on OCs-derived apoptotic bodies binds to salivated toll-like receptor 2 (TLR2), resulting in reduced initial activation of CD8 + T cells. Infusion of apoptotic bodies significantly increases the proliferation and spread of tumor cells in mice lacking apoptosis. In contrast, secondary lung metastasis does not occur in mice lacking Siglec-15. In addition, treatment with a specific monoclonal antibody against Siglec-15 (S15 NAB) significantly reduces the additional metastasis rate and improves survival in mice with advanced breast cancer and bone metastases (66).

**Table 1:** Siglec-15 Pan-Cancer analysis.

Cancer type	Gene expression levels	Sample size	Test method	Conclusion	Ref.
Colonic adenocarcinoma (COAD)	↑	102	IHC	Siglec-15 suppresses immunity by decreasing CD8 TILs in COAD	(48)
Esophageal carcinoma (ESCA)	↑	130	IHC,RT-qPCR	The presence of Siglec-15 in esophageal squamous cell carcinoma correlates with an increased rate of pathological complete response and improved survival outcomes	(49)
Pancreatic cancer (PAAD)	↑	209	mfIHC, IHC, ELISA, WB, RT-qPCR, FCM	Blocking SIGLEC-15 in pancreatic cancer may attenuate impaired T-cell infiltration and enhance antitumor activity	(50)
Liver hepatocellular carcinoma (LIHC)	↑	221	RT-qPCR, WB, CCK8, FCM, scRNA-seq, IHC	SIGLEC-15 can promote tumor immune escape by inducing CD8 + T cell apoptosis in HCC	(51)
Adenocarcinoma of lung (LUAD)	↑	135	IHC, Mihc, *RT‐qPCR*	PD-L1 independent Siglec-15TAM suppresses the immune microenvironment in patients with nonmetastatic LUAD	(52)
Adenocarcinoma of lung (LUAD)	↑	213	mfIHC, MSI	Siglec-15 positively correlates with CD8 T cell and tumor-associated macrophages (TAMs) infiltration in LUAD	(53)
Head and neck squamous cell carcinoma (HNSC)	↑	69	LC-MS, GC-MS, RNA-seq, CCK8, CFU, EdU, WB, ChIP, IF, *mIHC, RT‐qPCR, TIL, ELISA, FCM, IHC*	Tumor-derived Kyn promotes depletion of CD8 T cells in HNSCC via the AhR/Siglec-15 pathway	(54)
Thyroid cancer (THCA)	↑	164	IHC	Elevated expression of Siglec-15 mRNA facilitate extrathyroidal extension and lymph node metastasis, while patients with high Siglec-15 expression experience immune dysfunction	(55)
Bladder cancer	↑	63	RNA-seq, *RT‐qPCR*	Siglec-15 may modulates the non-inflammatory tumor microenvironment and may be biomarker for predicting the molecular subtypes of bladder cancer	(56)
Brain and central nervous system cancers	↑	101	IHC, IF	Siglec-15 expression on glioma cells and immune cells may mediate immune escape through cell adhesion	(57)
Colorectal cancer	↑	805	IHC	Siglec-15 protein is highly expressed in CRC compared to PD-L1	(58)
Osteoma sarcomatosum	↑	52	RNA-seq, WB, Co-IP, TEM, IHC,IF	Autophagy stimulated by Siglec-15 enhances migration and invasion by acting on the Beclin-1/ATG14 pathway and triggering epithelial-mesenchymal transition (EMT) both *in vitro* and *in vivo*	(59)
Stomach cancer	——	71	IHC	Siglec-15 did not detect significant differences in gastric cancer	(60)

↑, up-regulation.

### 5.3 Clinical trials of Siglec-15 in cancer

The Siglec-15 inhibitors are under vigorous development. The humanized monoclonal antibody (NC318) was initially recognized as the sole Siglec-15 inhibitor in 2019 to be utilized in clinical settings. This inhibition is undergoing phase I trials for treating solid tumors (67). Notably, no dose-limiting toxicities were observed at various cross-dose levels throughout the study (68).

Dr. Anthony Tolcher indicated that NC318 demonstrated safety and good tolerability among 49 individuals with various types of cancers, including NSCLC. NC318 is currently undergoing a Phase II assessment for NSCLC, ovarian cancer, head and neck cancer, and triple-negative breast cancer. The Phase II efficacy outcomes reported the objective response rate (ORR) for NC318 (monotherapy) of 4.8% (4 of 83), with a complete response (CR) at 1.2% (1 of 83) and a disease control rate (DCR) of 38% (32 of 83), regardless of PD-L1 or Siglec-15 expression status. While the median duration of response is unknown, two patients (achieving CR and PR) have been treated for over 2 years with no indications of disease progression. Shum et al. (68) discovered that the effectiveness of NC318 is correlated with dose-dependent increases in soluble Siglec-15 levels, indicating that it is a promising biomarker for assessing the impact of NC318. Furthermore, studies have revealed that the expression of Siglec-15 on cancer cell surfaces in screen-based biopsy specimens is associated with progression-free survival (PFS) and treatment duration. Phase II trials showed more promising results than Phase I trials, suggesting that targeted therapies related to Siglec-15 may offer more extensive research opportunities in the future, potentially facilitating advancements in other targeted therapies. Additionally, immunotherapy targeting PD-1 holds significant potential for success.

## 6 Siglec-15 in hematologic diseases

**Figure 5:**
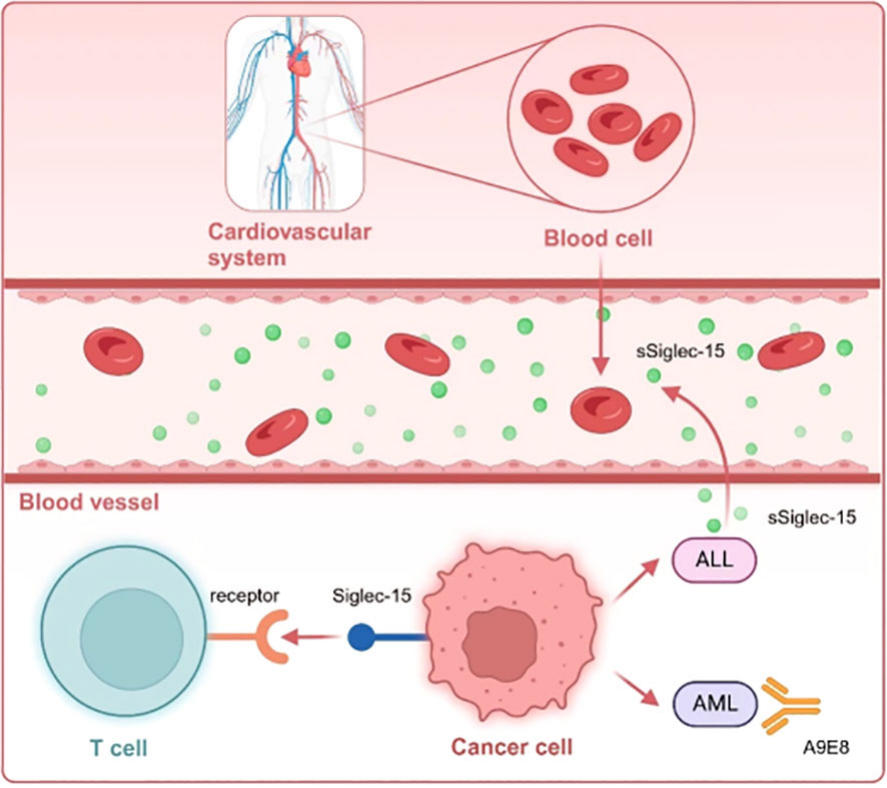
Siglec-15expressed on TAM or hematologic tumor cells interacts with unknown receptors on T cells. Notably, the Siglec-15 protein is secreted as a soluble form (sSiglec-15) from the plasma membrane into the bloodstream in the context of ALL, regulating the immune microenvironment. Moreover, the monoclonal antibody A9E8 targeting Siglec-15, designed from AML, need to be further investigated for potential drug development.

While shifting from immune enhancement to the tumor immune microenvironment (TIME) and normalization can advance immunotherapy (IO) in solid tumors, immune enhancement is the primary treatment approach for hematologic malignancies. This is primarily due to its potential to overlook the extent of immune “deficiencies” that need to be normalized. Although Siglec-15 can facilitate the normalization of immunotherapy for solid tumors, the related study on hematologic disorders is still in the early stages (**Figure 5**).

### 6.1 Acute myeloid leukemia

#### 6.1.1 Current status of immunotherapy research in AML

Acute myeloid leukemia (AML) develops from myeloid stem or progenitor cells. AML is marked by the malignant proliferation of cells, clonal evolution, and genetic diversity and mainly affects adults. Notably, the likelihood of developing AML increases as individuals age, making older populations more susceptible to the disease (69). The successful cure rates of AML are low, with over half of patients experiencing relapses, indicating a significant incidence of treatment failures. This is largely due to the persistent presence of leukemic stem cells (LSCs) that remain immune to common radiation treatment when in a dormant state. Notably, overcoming drug resistance is a considerable challenge (70, 71).

CAR-T therapy continues to show limited effectiveness in treating AML. The potential targets for CAR-T in AML consist of CD123, CD33, NKG2D, Lewis Y, FLT-3, CLL-1, CD44v6, IL1-RAP, and TIM-3 (72, 73). However, CD123 is the most studied target. Budde conducted CAR-T cell treatment in AML patients focused on CD123 and reported positive outcomes in several individuals (74). CD70-CAR T cells in AML patients who are positive for CD70 have demonstrated considerable targeting or tumor toxicity towards normal hematopoietic stem cells and myeloid progenitor cells (75). The myeloid lineage in AML patients exhibits heightened immunosuppression due to the near depletion and functional impairment of effector T cells and NK cells. This situation is further compounded by an enhanced prevalence of hyperfunctional regulatory T cells (Tregs) (76). Additionally, PD-1/PD-L1 signaling is related to disease progression in leukemia, playing a role in the suppression of host anti-tumor immune feedback. This results in the failure of CD8+ T cells, thus facilitating Treg-mediated inhibition of effector T cells (77). Although PD-1/PD-L1 levels are elevated in AML individuals, AML treatment with PD-1/PD-L1 inhibitors is associated with a significant risk of relapse in prognosis, and does not eliminate the possibility of inducing other neoplastic conditions. The expression of numerous immune checkpoints, including LAG-3, TIM-3, B7-H3, B7-H4, and VISTA (PD-1H), is low in AML. Additionally, although CD33 is found in about 90% of AML patients as a surface marker, studies have demonstrated that CD33, CD123, and CLL-1 antibodies are effective in AML treatment with significant adverse effects. The absence of AML-specific targets indicates that methodologies relying on non-specific antigen targets lead to considerable myelotoxicity and off-target side effects, posing substantial challenges for clinical trials. As a result, the advancement of immunotherapy for AML has been limited.

#### 6.1.2 Siglec-15 in acute myeloid leukemia

Siglec-15 is an antigen located outside cells, including AML cells, thus is a promising therapeutic target for AML. Huan et al. developed a monoclonal antibody, A9E8, to specifically attach to Siglec-15 utilizing phage display technology (78). Furthermore, researchers discovered that A9E8, a highly specific monoclonal antibody, can identify and bind to both overexpressed cell surface Siglec-15 and endogenous Siglec-15 in leukemia cell lines, including K562. Moreover, A9E8 can label Siglec-15 present on CD33 + outer blood mononuclear cells from AML patients. Conversely, the expression of Siglec-15 is extremely low in CD34 + stem cells derived from healthy donors and in most mature circulating leukocytes, macrophages, and dendritic cells cultured *in vitro* or sourced from non-AML donors. This finding implies that targeting Siglec-15 may provide a more significant therapeutic advantage than targeting CD33. Moreover, A9E8 can label CD34 + leukemic stem cells (LSCs) in AML. Nevertheless, peripheral blood cells from non-AML patients do not exhibit A9E8 staining, after granulocyte colony-stimulating factor (G-CSF) stimulation. The A9E8 antibody demonstrates significant specificity within the AML bone marrow environment, making it a valuable target for further research. These findings indicate that Siglec-15 has a rapid internalization capability, primarily due to its cytosolic ITSM motif, with a half-life of 174 seconds when cross-linked with A9E8 (78). This characteristic may aid in designing toxin-conjugated antibodies, specifically targeting Siglec-15-positive AML cells. Such a rapid internalization mechanism can enhance the transportation of drugs, toxins, or radioactive agents into the cells, thereby increasing therapeutic effectiveness within tissues.

Nevertheless, this study has some limitations. First, the initial sample size is small, necessitating a large sample size to confirm the reliability and generalizability of the findings. Additionally, further investigations should examine the expression patterns of Siglec-15 in AML patients compared with healthy donors since there is a lack of extensive experimental validations to assess its potential as a targeted therapy. Finally, further clinical trials are needed to validate the effectiveness and safety of A9E8 in treating AML. Although significant advancements have been made in the research of monoclonal antibodies targeting Siglec-15, including A9E8. Additional fundamental experimental and clinical investigations are necessary to better understand their therapeutic potential, mechanisms of action, safety profiles, and efficacy. This research highlights the crucial role of Siglec-15 in AML, suggesting a new pathway that may also improve personalized treatment plans for AML patients, enabling the selection of more suitable therapeutic options. Therefore, related studies may offer valuable insights and guidance for research on immunotherapy in different cancer types.

### 6.2 Acute lymphoblastic leukemia

#### 6.2.1 Current status of immunotherapy research in ALL

Acute lymphoblastic leukemia (ALL) is a blood cancer characterized by the unusual spreading of B-lineage, or T-lineage cells stemming from lymphocytes within the bone marrow (79). ALL is common in pediatric patients and is mainly treated with chemotherapy. Notably, many chemotherapeutic agents are irreversible for young children and may lead to organ damage during treatment, thus immunotherapy, which causes less harm, is prioritized. The development of CAR-T therapy for addressing hematological cancers, particularly ALL and B-cell lymphoma expressing CD19 has significantly progressed (80, 81). CAR-T cells targeting CD19 in B-cell tumors have introduced a new phase of engineered cancer immunotherapy (82), facilitating deep and complete remissions. This method may offer long-lasting treatment for several patients resistant to chemotherapy. Nevertheless, about 10%-30% of patients do not attain remission, and over 50% of those receiving CD19-targeted CAR-T therapy experience a relapse (83). The US Food and Drug Administration (FDA) has authorized two CAR T-cell therapies targeting CD19 for treating relapsed or refractory (r/r) B-ALL (tisagenlecleucel (Kymriah) and brexucabtagene autoleucel (Tecartus)). Tisagenlecleucel (Kymriah)features the 4-1BB coactivation domain and received FDA approval in 2017 for use in pediatric and adolescent patients aged 25 years and below (84, 85). Brexucabtagene autoleucel (Tecartus) features the CD28 coactivation domain, and was approved in 2021 for r/r B-ALL patients aged 18 years and older (86). Although less than 50% of pediatric patients who receive tisagenlecleucel achieve long-term event-free survival (EFS) (87), the response rate of CD19 CAR T cell therapy in B-cell acute lymphoblastic leukemia (B-ALL) is remarkable. In contrast, T-cell acute lymphoblastic leukemia (T-ALL) exhibits a subpar therapeutic response due to its constrained structure and associated outcomes. Overall, ALL patients demonstrate a higher complete response (CR) rate and may be cured without additional treatment. Furthermore, future studies should assess the mechanisms of immune evasion that are crucial for disease advancement and effective strategies to enhance therapeutic immune regulation in lymphocytic cancers.

#### 6.2.2 Siglec-15 in acute lymphoblastic leukemia

Pillsbury et al. (23) indicated that Siglec-15 expression is elevated in samples from B-ALL and AML patients compared with peripheral blood mononuclear cells (PBMCs) obtained from healthy individuals based on Oncomine database analyses. Furthermore, the expression and localization of Siglec-15 in B-ALL are modulated by the NF-κB signaling pathway. NF-κB activation results in increased external presence of Siglec-15 on B-ALL cells. Soluble Siglec-15 (sSiglec-15) is positively correlated with various immunosuppressive cytokines in the plasma of B-ALL patients, such as MCP-1/CCL2 and IL-6, and negatively correlated with cytokines with anti-leukemic properties, such as IL-12. These findings indicate that Siglec-15 may shape the immune microenvironment in B-ALL. Researchers have also found that Siglec-15 can directly inhibit the early activation of Jurkat cells expressing CD19-CAR using a recombinant Siglec-15 protein. Moreover, a study showed that Siglec-15 gene can be successfully knocked out in a murine model using shRNA and CRISPR/Cas9 techniques. Siglec-15-deficient B-ALL cells cannot proliferate rapidly in immunocompetent mice, leading to a significant reduction in leukemia burden and a marked extension of survival in the mice. Moreover, Siglec-15-null B-ALL cells elicit a more vigorous anti-leukemia immune response, characterized by the proliferation of CD8+ T cells, enhanced T cell activation, and degranulation. These findings suggest that Siglec-15 plays a critical role in the immune evasion mechanisms of B-ALL.

However, the research has some limitations: Firstly, the specific mechanism through which Siglec-15 operates in B-ALL, including its impact on T cell activity, is not thoroughly investigated. Second, the investigation lacks data regarding the specific expression levels of Siglec-15 in B-ALL individuals, which could influence the assessment of its clinical significance. Finally, the study only focused on B-ALL. Therefore, further studies should determine whether analogous mechanisms exist in other types of leukemia. Nonetheless, the study holds significant importance regarding the pathogenesis and immune evasion mechanisms of B-ALL within the contemporary medical research landscape. Therefore, the study provides a basis for innovative treatment approaches and investigations related to B-ALL, offering new insights into potential therapeutic targets. These findings indicate the critical role of Siglec-15 in B-ALL, establishing a robust theoretical foundation for developing treatment strategies centered on Siglec-15. Furthermore, the findings provide considerable reference value and inspiration for exploring other forms of leukemia and diseases associated with immune evasion, thereby facilitating the advancement of scientific inquiry and clinical applications in relevant fields.

### 6.3 Lymphadenoma

#### 6.3.1 Current status of immunotherapy research in lymphadenoma

Lymphoma is a malignant tumor that arises from lymphoid tissue. Lymphoma is primarily categorized into two main types: Hodgkin lymphoma (HL) and non-Hodgkin lymphoma (NHL). The B-cell acute lymphoblastic leukemia or lymphoma is widely treated with CD19 CAR-T, which currently achieves an impressive cure rate with a low likelihood of relapse. Nevertheless, the prognosis for people with slipped back, or refractory (r/r) hematologic cancer remains largely poor, especially in instances of relapsed or refractory hostile fully grown B-cell lymphomas, such as diffuse large B-cell lymphoma (DLBCL) and Burkitt lymphoma (BL) (88, 89). The percentage of pediatric NHL attributed to DLBCL is about 10-20%, making it the most prevalent subtype in adults. NHL has a considerable cure rate, with a five-year event-free survival exceeding 75%. Nonetheless, about 30-40% of adults diagnosed with DLBCL who undergo initial treatment may encounter refractory or relapsing disease. Managing r/r DLBCL poses a significant clinical challenge, even with aggressive treatment protocols. As a result, therapies, such as CD22/SIGLEC2 CAR-T and CD33/SIGLEC3 CAR-T, have been introduced to address refractory leukemia or lymphoma (73). In a clinical trial, CD19/22 dual-target CAR-T (AUTO3) combined with pembrolizumab therapy for refractory large B-cell lymphoma (NCT03289455), demonstrated a total reaction effect of 66%, including a complete response (CR) rate of 48.9% and a partial response (PR) rate of 17% (90). The Siglecs family members can improve lymphoma treatment.

#### 6.3.2 Siglec-15 in lymphadenoma

Although only a few studies have explored the role of Siglec-15 in the context of hematological disorders, advancements have been made regarding other Siglec family members. For instance, Siglec-3 is predominantly expressed on the surface of AML cells, but it is absent in normal hematopoietic stem cells, making it a potential target for AML therapy (91). Furthermore, Siglec-3 has been detected on the cell surface in cases of myelodysplastic syndrome and ALL (92). Besides, Siglec-6 is expressed in chronic lymphocytic leukemia (CLL), mucosa-associated lymphoid tissue lymphoma, and blasts from AML (93). Notably, Siglec-6 mRNA and protein are not present in hematopoietic stem cells, indicating that it is a promising target for immunotherapy in CLL (94). Alemtuzumab, a humanized monoclonal antibody targeting Siglec-10, and other treatments for CLL are currently being evaluated in clinical studies (95). Several investigations have indicated that the expression levels of Siglec-1, CD22, and Siglec-14 are significantly reduced in AML patients with FLT3 mutations, while the levels of CD33 and Siglec-15 are markedly increased (96). Michael et al. identified Siglec-7 as a promising therapeutic target in multiple myeloma (97). Additionally, the role and functionality of Siglec-15 are inadequately characterized, necessitating further exploration of its potential use in hematologic disorders.

## 7 Future research and prospects

Immunotherapy in oncology has recently attracted much attention, achieving remarkable results. However, the use of immunotherapy in the treatment of hematological diseases is limited due to the lack of specific target antigens and the related effects on normal bone marrow function. Siglec-15 is a unique immune checkpoint that can serve as a critical target for cancer intervention. To date, studies have highlighted the important role of Siglec-15 in regulating osteoclast activity. Besides, osteoclasts are related to myeloid cells. Osteoclasts and tumor-infiltrating myeloid cells originate from common precursor cells. The adaptive proteins, such as DAP12 and FcRγ, are highly expressed in myeloid-derived cells, thereby allowing Siglec-15 to bind silicic acid and promoting intracellular signal transduction. This mechanism may reveal the potential role of Siglec-15 as a receptor or ligand in hematological diseases. Nonetheless, further investigation of the therapeutic potential is needed. However, many hematological diseases have relatively low mutation rates and limited availability of new antigens compared with solid tumors. Although Siglec-15 has shown considerable results in solid tumor immunotherapy, further studies should explore the pathogenesis of Siglec-15 in hematological diseases and developing potent antibodies to establish more reliable clinical treatment strategies. Besides, future studies should further clarify the specific mechanism of action of Siglec-15 in different hematologic diseases, explore its interaction network with other molecules, and develop more effective diagnostic and therapeutic strategies. Meanwhile, large-scale clinical studies are needed to validate the feasibility and efficacy of Siglec-15 as a biomarker and therapeutic target. In conclusion, Siglec-15, as an emerging field of research, provides new perspectives and opportunities for understanding the pathogenesis of hematologic diseases and developing new treatments. Therefore, Siglec-15 may play a key role in the diagnosis and treatment of hematological diseases.
